# Supplementary material for: Dynamics of the 4D genome during in vivo lineage specification and differentiation
Source: Nat Commun. 2020 Jun 1;11:2722. doi: 10.1038/s41467-020-16598-7 (PMC7264236; doi:10.1038/s41467-020-16598-7)
Supplement: Supplementary file 3 — Reporting Summary [file 41467_2020_16598_MOESM3_ESM.pdf]

natureresearch

Corresponding author(s): Jim R. Hughes and Douglas R. Higgs

Last updated by author(s): Apr 16, 2020

Reporting Summary

Nature Research wishes to improve the reproducibility of the work that we publish. This form provides structure for consistency and transparency in reporting. For further information on Nature Research policies, see Authors & Referees and the Editorial Policy Checklist .

Statistics

For all statistical analyses, confirm that the following items are present in the figure legend, table legend, main text, or Methods section.

n/a	Confirmed	
☐	☒	The exact sample size (*n*) for each experimental group/condition, given as a discrete number and unit of measurement
☐	☒	A statement on whether measurements were taken from distinct samples or whether the same sample was measured repeatedly
☐	☒	The statistical test(s) used AND whether they are one- or two-sided<br>*Only common tests should be described solely by name; describe more complex techniques in the Methods section.*
☒	☐	A description of all covariates tested
☒	☐	A description of any assumptions or corrections, such as tests of normality and adjustment for multiple comparisons
☐	☒	A full description of the statistical parameters including central tendency (e.g. means) or other basic estimates (e.g. regression coefficient) AND variation (e.g. standard deviation) or associated estimates of uncertainty (e.g. confidence intervals)
☐	☒	For null hypothesis testing, the test statistic (e.g. *F*, *t*, *r*) with confidence intervals, effect sizes, degrees of freedom and *P* value noted<br>*Give P values as exact values whenever suitable.*
☒	☐	For Bayesian analysis, information on the choice of priors and Markov chain Monte Carlo settings
☒	☐	For hierarchical and complex designs, identification of the appropriate level for tests and full reporting of outcomes
☒	☐	Estimates of effect sizes (e.g. Cohen's *d*, Pearson's *r*), indicating how they were calculated

Our web collection on statistics for biologists contains articles on many of the points above.

Software and code

Policy information about availability of computer code

Data collection	Next-generation sequencing (Illumina). No software was used for data collection.
Data analysis	HiCPro, CCseqBasic, NGseqBasic v20, BEDTools v2.25.0, Cell Ranger v2.1.1, CITE-seq-count v0.2, Scrublet v0.2, Seurat v2, MACS2 v2.0.10.20131216, . Custom codes available in https://github.com/Hughes-Genome-Group/CCseqBasicF/releases, https://github.com/oudelaar/TiledC, https://github.com/rbeagrie/alpha-tiledc.

For manuscripts utilizing custom algorithms or software that are central to the research but not yet described in published literature, software must be made available to editors/reviewers. We strongly encourage code deposition in a community repository (e.g. GitHub). See the Nature Research guidelines for submitting code & software for further information.

Data

Policy information about availability of data

All manuscripts must include a data availability statement. This statement should provide the following information, where applicable:

- Accession codes, unique identifiers, or web links for publicly available datasets
- A list of figures that have associated raw data
- A description of any restrictions on data availability

Tiled-C, ATAC-seq and single-cell RNA-seq data generated in this study have been deposited in the Gene Expression Omnibus (GEO) under accession code GSE137477. All RNA-FISH image files are archived in the Imaging Data Resource, and the scoring of images that underlies figure 3e is provided in the source data file. A UCSC hub for visualizing ATAC-seq and single-cell RNA-seq mean expression data is available at http://sara.molbiol.ox.ac.uk/public/hugheslab/alpha-tiledc/hub.txt.

Field-specific reporting

Please select the one below that is the best fit for your research. If you are not sure, read the appropriate sections before making your selection.

- [x] Life sciences
- [ ] Behavioural & social sciences
- [ ] Ecological, evolutionary & environmental sciences

For a reference copy of the document with all sections, see nature.com/documents/nr-reporting-summary-flat.pdf

Life sciences study design

All studies must disclose on these points even when the disclosure is negative.

Sample size	We did not perform sample size calculation. Experiments were performed in 2-3 biological replicates as is common in the field and the observed biological effects of interest were very robust between replicates.
Data exclusions	No data exclusions.
Replication	Experiments were performed in 2-3 independent biological replicates as described and all attempts were successful.
Randomization	Cells were allocated to different groups using FACS analysis. Control of covariates was not relevant for our study as the different groups represent different cell populations of which multiple characteristics were analyzed.
Blinding	FISH image analysis was blinded. Blinding was not relevant for the analysis of next-generation sequencing data in this study, as all samples were analyzed using the exact same pipelines and scripts.

Reporting for specific materials, systems and methods

We require information from authors about some types of materials, experimental systems and methods used in many studies. Here, indicate whether each material, system or method listed is relevant to your study. If you are not sure if a list item applies to your research, read the appropriate section before selecting a response.

Materials & experimental systems

n/a	Involved in the study	
[ ]	[x]	Antibodies
[ ]	[x]	Eukaryotic cell lines
[x]	[ ]	Palaeontology
[ ]	[x]	Animals and other organisms
[x]	[ ]	Human research participants
[x]	[ ]	Clinical data

Methods

n/a	Involved in the study	
[x]	[ ]	ChIP-seq
[ ]	[x]	Flow cytometry
[x]	[ ]	MRI-based neuroimaging

Antibodies

Antibodies used	Biotin-conjugated anti-Ter119 (BD 553672, clone TER119), PE-Cy7-conjugated anti-CD71 (BioLegend 113811, clone RI7217), panel of 5 FITC-conjugated lineage antibodies (anti-CD41 BD 553848 clone MWReg30, anti-CD45R BD 553087 clone RA3-6B2, anti-CD3e BD 553061 clone 145-2C11, anti-CD11b BD 557396 clone M1/70 and anti-Ly-6G/6C BD 553126 clone RB6-8C5), anti-Ter119 (BioLegend 116241 clone TER119) conjugated to a DNA oligonucleotide (ADT2 GAGGCGATTGAT), panel of 9 DNA-oligonucleotide conjugated antibodies (anti-CD71: BioLegend 113802 [clone RI7217] conjugated with ADT9 [CGAAGAAGGAGT], anti-CD41: BioLegend 133919 [clone MWReg30] conjugated with ADT3 [TGTCCGGCAATA], anti-CD45R: BioLegend 103249 [clone RA3-6B2] conjugated with ADT5 [GATCGTAATACC], anti-CD3e: BioLegend 100345 [clone 145-2C11] conjugated with ADT7 [CATCGGTGTACA], anti CD11b: BioLegend 101249 [clone M1/70] conjugated with ADT1 [CATGATTGGCTC], anti-Ly6G/6C: BioLegend 108449 [clone RB6-8C5] conjugated with ADT4 [TGGTGAACCTGG], anti-CD44: BioLegend 103051 [clone IM7] conjugated with ADT8 [GTCTAGACTTCG], anti-cKit: BioLegend 105829 [clone 2B8] conjugated with ADT6 [AAGCGCTTGGCA], non-specific IgG conjugated with ADT10 [CGGAGTAGTAAT]), sheep anti-DIG FITC (Roche), rabbit anti-sheep FITC (Vector).
Validation	All antibodies were validated by their manufacturers for the application (flow cytometry) and species (mouse) used in this study. Furthermore, all antibodies used have been individually titrated prior to use to identify their optimal concentration in the required application. All experiments included fluorescence-minus-one (FMO) controls.

Eukaryotic cell lines

Policy information about cell lines

Cell line source(s)	Mouse ES cells ES-E14TG2a.4 and A9. These cell lines have been maintained in our in-house facility for 3 decades and were originally obtained from the University of Edinburgh via Andrew Smith.

Authentication	The cells were not authenticated by standardized methods, but are routinely karyotyped and tested for contamination and infection.
Mycoplasma contamination	Routinely tested within our facility and negative.
Commonly misidentified lines (See ICLAC register)	No commonly misidentified cell lines were used in our study.

Animals and other organisms

Policy information about studies involving animals; ARRIVE guidelines recommended for reporting animal research

Laboratory animals	Mus musculus, C57BL/6, female, 3-9 months old.
Wild animals	Our study did not involve wild animals.
Field-collected samples	Our study did not involve samples collected from the field.
Ethics oversight	Experimental procedures were in accordance with the European Union Directive 2010/63/EU and/or the UK Animals (Scientific Procedures) Act (1986) and protocols were approved through the Oxford University Local Ethical Review process.

Note that full information on the approval of the study protocol must also be provided in the manuscript.

Flow Cytometry

Plots

Confirm that:

- [x] The axis labels state the marker and fluorochrome used (e.g. CD4-FITC).
- [x] The axis scales are clearly visible. Include numbers along axes only for bottom left plot of group (a 'group' is an analysis of identical markers).
- [x] All plots are contour plots with outliers or pseudocolor plots.
- [x] A numerical value for number of cells or percentage (with statistics) is provided.

Methodology

Sample preparation	Primary erythroid progenitor cells were isolated from fetal livers freshly isolated at e12.5-e13.5 from C57BL/6 mouse embryos. 5-15 livers were pooled together for each experimental replicate, mechanically dissociated in staining buffer (PBS, 0.2% BSA, 5mM Glucose) and strained through a 30-µm strainer. Cells were immunostained at 4 °C in the presence of rabbit IgG (200 µg/ml, Jackson Laboratories 015-000-003) to block Fc receptors. To enrich for early erythroid progenitors, cells were first stained with 5 µg/ml biotin-conjugated anti-Ter119 (BD 553672) for 30 minutes, before magnetic depletion using streptavidin nanobeads (BioLegend Mojosort 480016) following the manufacturer's instructions. Cells were then incubated with 0.5 µg/ml APC-conjugated streptavidin (BD 553672), 0.33 µg/ml PE-Cy7-conjugated anti-CD71 (BD Biosciences) and a panel of 5 FITC-conjugated lineage antibodies (anti-CD41, anti-CD45R, anti-CD3e, anti-CD11b and anti-Ly-6G/6C, all at 1 µg/ml; BD 553848, 553087, 553061, 557396 and 553126, respectively) for 45 minutes. Cells were then resuspended in FACS running buffer (staining buffer plus 2 mM EDTA). 0.66 µg/ml Hoechst was added immediately prior to sorting in order to distinguish live cells.
Instrument	BD FACSAria™ Fusion.
Software	Samples were collected using BD FACSDiva™ and plots prepared using FlowJo.
Cell population abundance	Small aliquots of post-sort fractions were re-run on the cytometer immediately after sorting to determine purity. Purities of all fractions were consistently >90%.
Gating strategy	Cells gated on FSC-A/SSC-A, then single cells on FSC-A/FSC-W, then live cells on FSC-A/DAPI, then Lin- cells on FSC-A/FITC, then populations determined by gating on PE-Cy7/APC. A figure giving the gating for PE-Cy7/APC is provided. The prior gates are standard gates that have been described in previous publications, as detailed in the paper's reference list.

- [x] Tick this box to confirm that a figure exemplifying the gating strategy is provided in the Supplementary Information.
