# Supplementary material for: Dynamics of the 4D genome during in vivo lineage specification and differentiation
Source: Nat Commun. 2020 Jun 1;11:2722. doi: 10.1038/s41467-020-16598-7 (PMC7264236; doi:10.1038/s41467-020-16598-7)
Supplement: Supplementary file 2 — Peer Review File [file 41467_2020_16598_MOESM2_ESM.pdf]

Editorial Note: This manuscript has been previously reviewed at another journal that is not operating a transparent peer review scheme. This document only contains reviewer comments and rebuttal letters for versions considered at *Nature Communications*.

REVIEWERS' COMMENTS:

Reviewer #2 (Remarks to the Author):

Authors have made a thorough revision, incorporating new material and fulfilling reviewers requests.

Reviewer #3 (Remarks to the Author):

I am happy with the responses of the authors to my criticisms.

REVIEWERS' COMMENTS:

Reviewer #2 (Remarks to the Author):

Authors have made a thorough revision, incorporating new material and fulfilling reviewers requests.

Thank you.

Reviewer #3 (Remarks to the Author):

I am happy with the responses of the authors to my criticisms

Thank you.
